# Effect of Mesoporous Zinc Oxide Nanoparticle Incorporation on the Bond Strength of Resin-Modified Glass Ionomer Cement to Enamel and Dentin: An In Vitro Study

**DOI:** 10.1155/ijod/8406448

**Published:** 2025-09-16

**Authors:** Zahra Jowkar, Melika Kiumarsi, Seyed Ahmadreza Hamidi, Ali Moaddeli

**Affiliations:** ^1^Oral and Dental Disease Research Center, Department of Operative Dentistry, School of Dentistry, Shiraz University of Medical Sciences, Shiraz, Iran; ^2^Department of Operative Dentistry, School of Dentistry, Shiraz University of Medical Sciences, Shiraz, Iran; ^3^Department of Oral and Maxillofacial Surgery, School of Dentistry, Shiraz University of Medical Sciences, Shiraz, Iran; ^4^Legal Medicine Research Center, Legal Medicine Organization, Tehran, Iran

**Keywords:** bond strength, mesoporous, resin-modified glass ionomer cement, zinc oxide nanoparticles

## Abstract

**Objective:** The purpose of this in vitro study was to evaluate the microshear bond strength (µSBS) of resin-modified glass ionomer cement (RMGIC) to enamel and dentin, with and without the inclusion of zinc oxide nanoparticles (ZnO NPs) and mesoporous ZnO NPs.

**Materials and Methods:** 140 extracted human third molars were used, categorized into two primary groups based on the substrate—enamel or dentin—and further divided into seven subgroups (*n* = 10). The groups consisted of RMGIC alone, and RMGIC enhanced with either 3%, 5%, or 7% of conventional ZnO NPs or mesoporous ZnO NPs. µSBS testing was performed, followed by statistical evaluation.

**Results:** The type of substrate and the incorporation of NPs significantly affected bond strength (*p* < 0.001), with enamel showing superior µSBS compared to dentin (*p* < 0.001). The subgroup containing 5% mesoporous ZnO NPs demonstrated the highest bond strength overall (*p* < 0.001), while no significant variations were detected among the other groups (*p* > 0.05).

**Conclusion:** Enhancing RMGIC with 5 wt.% mesoporous ZnO NPs markedly improved adhesion to both enamel and dentin. Increasing the (nanoparticle) NP concentration beyond 5% did not further enhance bonding performance. Enamel exhibited consistently better bonding than dentin in all subgroups. These results highlight the potential of 5% mesoporous ZnO NPs in improving the clinical efficacy of RMGIC.

## 1. Introduction

Glass ionomer cements (GICs) are widely used in restorative dentistry due to their fluoride release and chemical adhesion to dental tissues [1]. GICs offer advantages, such as sustained fluoride release, biocompatibility, esthetic tooth-like appearance, and thermal compatibility with tooth structure [1, 2]. However, their application is limited by low mechanical strength, moisture sensitivity during setting, and poor wear resistance, restricting their use to low-stress clinical areas [2, 3].

To address these shortcomings, several materials have been incorporated into GICs to improve their physical performance [4, 5]. Resin-modified GICs (RMGICs), developed through the addition of resin monomers, show enhanced diametral tensile, flexural, and compressive strength and allow light-curing, providing improved handling and control during placement [6, 7]. The inclusion of resin shortens the setting duration, decreases sensitivity to moisture, provides longer working time, and improves both translucency and overall esthetics [7, 8].

In restorative dentistry, preventing bacterial colonization after caries removal is critical for restoration longevity. Incorporating antibacterial agents into restorative materials helps inhibit bacterial growth and penetration, thereby reducing the risk of recurrent caries [9, 10]. Although, GICs exhibit antibacterial effects attributed to fluoride release and low initial pH, they may not provide sufficient long-term protection against cariogenic bacteria, potentially leading to secondary caries and restoration failure [11]. Consequently, enhancing the antibacterial properties of GICs remains a focus of ongoing research to improve their clinical performance and durability.

Zinc oxide (ZnO), known for its antimicrobial properties, is commonly used in dental materials [10, 12]. It is affordable, stable, and biocompatible [10]. Recently, ZnO nanoparticles (ZnO NPs) have gained popularity due to their enhanced antibacterial efficacy [10]. Due to their small size, NPs penetrate dentinal tubules more effectively than larger particles [10]. ZnO NPs show significant antibacterial activity against *S. mutans* and *Lactobacillus*, inhibiting biofilm formation when added to dental materials [13]. Moreover, they improve bond strength in enamel and dentin without compromising it during pretreatment [14].

Mesoporous materials, with pore sizes ranging from 2 to 50 nm, have gained significant interest in both medicine and dentistry [15]. These materials are valued for their adjustable pore sizes, biocompatibility, high surface area, and lack of toxicity [16]. Their pore structure can be easily modified, and various synthesis methods enable the optimization of their composition, structure, and porosity [16]. Mesoporous ZnO NPs, characterized by their large surface area, porosity, crystallinity, and antimicrobial properties, show promising potential for a wide range of therapeutic applications [15, 16].

Nanosized materials, particularly mesoporous ZnO NPs, demonstrate significantly enhanced antibacterial properties compared to their bulk counterparts, owing to their increased surface area-to-volume ratio, making them promising candidates as nanofillers for RMGICs [16]. Incorporating nanofillers, such as mesoporous ZnO NPs may enhance antibacterial activity and mechanical performance; however, preserving adequate bond strength is critical and must be thoroughly evaluated. Although, these materials show potential, their influence on the bond strength of resin-modified glass ionomer cement (RMGIC) to enamel and dentin in permanent teeth remains unclear. Therefore, the present study aimed to evaluate and compare the microshear bond strength (µSBS) of RMGIC to enamel and dentin of permanent teeth restored with RMGIC, with and without the incorporation of mesoporous ZnO NPs and ZnO NPs. The null hypothesis proposed that there would be no significant difference in the bond strength of RMGIC to enamel and dentin regardless of the addition of mesoporous ZnO NPs or ZnO NPs.

## 2. Materials and Methods

This experimental study utilized 140 sound human third molars, free from caries, fractures, or enamel defects. The study protocol received approval from the Research and Ethics Committee of Shiraz University of Medical Sciences (IR.SUMS.DENTAL.REC.1403.009). All procedures complied with the ethical standards of the Declaration of Helsinki. Teeth were extracted for orthodontic indications, and written informed consent was obtained after participants were informed about the study's objectives and the use of their extracted teeth. All experimental steps were performed by a trained operator who was blinded to the group allocations. Following extraction, teeth were cleaned with a periodontal curette and stored in 0.5% chloramine-T at 4°C for no longer than 1 month before being used.

### 2.1. Sample Size Calculation

Before beginning the study, an a priori sample size calculation was performed using G^*⁣*^*∗*^^Power software (version 3.1.9.7; Heinrich-Heine-Universität Düsseldorf, Düsseldorf, Germany) to ensure sufficient statistical power. The effect size (*f* = 1.30) was derived from a previously published study [17]. Based on a power of 80% (*β* = 0.20) and a significance threshold of 0.05 (*α* = 0.05), the analysis indicated that at least nine specimens per subgroup would be needed to detect a significant difference. To enhance the reliability of the results and accommodate possible variability, 10 specimens were assigned to each subgroup. Consequently, each experimental group for both enamel and dentin consisted of 10 samples, leading to a total of 140 specimens across all groups.

### 2.2. Specimen Preparation

140 human molar crowns were used after separating them from their roots at the cementoenamel junction with a water-cooled, low-speed diamond saw (Mecatome T201 A, Presi, Grenoble, France). The roots were discarded, and the crowns were randomly assigned into two groups: 70 samples for testing bond strength to enamel, and 70 for evaluating adhesion to dentin.

To prepare enamel surfaces, a 0.5 mm-deep flat area was created in the center of the buccal surface of each crown using the cutting machine mentioned above. Each crown was then embedded in acrylic resin blocks (Acropars, Marlic Medical Industries Co., Tehran, Iran), positioning the buccal surface facing upward and level with the base. The enamel was polished gently with 320-grit silicon carbide abrasive paper (Starcke GmbH & Co. KG, Melle, Germany) to ensure surface uniformity. Enamel quality and flatness were examined using a stereomicroscope (Stemi 305, Carl Zeiss AG, Oberkochen, Germany).

For the dentin group, the occlusal enamel and superficial dentin layers were removed to expose flat mid-coronal dentin using the same saw, under continuous water cooling. The dentin surfaces were also embedded in acrylic blocks with the surface aligned parallel to the base. A smear layer was created by polishing the exposed dentin for 60 s with 320-grit silicon carbide paper. Finally, the surfaces were rinsed and lightly dried using an air–water spray syringe (DABI Atlante, São Paulo, Brazil).

Before bonding the RMGIC to the prepared enamel and dentin surfaces, a conditioning step was performed using GC Cavity Conditioner (GC Corporation, Tokyo, Japan) in accordance with the manufacturer's guidelines. The conditioner was applied to the tooth surface using a cotton pellet or applicator sponge. It was allowed to react for 10 s, after which the surface was thoroughly rinsed with water and gently blotted dry, leaving the surface moist but not desiccated.

### 2.3. Experimental Design and Grouping

**Figure 1:**
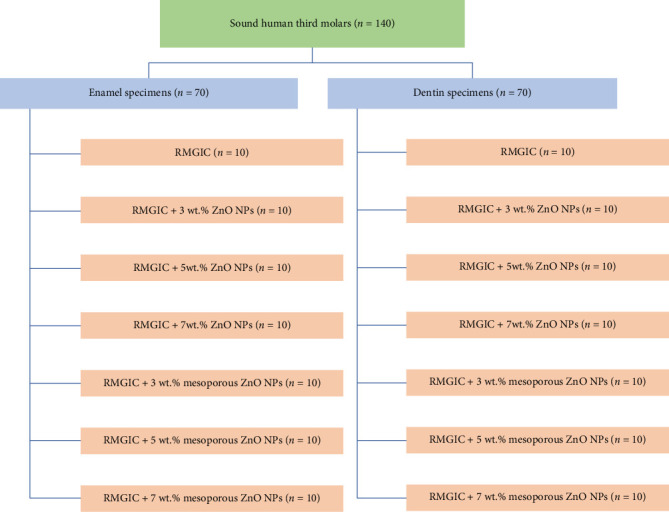
Schematic representation of the study design, detailing the different experimental groups, and the corresponding treatment protocols followed in each group.

The schematic layout of the experimental groups and protocols is illustrated in Figure 1. Two types of nanofillers were incorporated into the RMGIC: standard ZnO NPs, sourced from ASEPE Company (Tabriz, Iran), and mesoporous ZnO NPs synthesized and characterized as described in a previous study [16]. NPs were carefully weighed and thoroughly blended with the RMGIC powder using precise protocols, following a previously established method to ensure relatively uniform distribution [18]. Both nanoparticle (NP) powders were weighed with high precision to the nearest 0.001 g using a digital analytical balance (GR-3000, A & D CL Toshiba, Tokyo, Japan). These were then manually blended with the preweighed RMGIC powder (Fuji II LC, GC Corporation, Tokyo, Japan), composed of ~95% amorphous fluoroaluminosilicate glass and 5% polyacrylic acid. Each experimental group was prepared according to specific weight percentages of nanofillers as described in the subsequent sections.

A total of 140 samples (70 enamel and 70 dentin specimens) were randomly distributed into seven subgroups (*n* = 10 per group for both enamel and dentin):

• Group 1: RMGIC without any filler (control).
• Group 2: RMGIC + 3 wt.% ZnO NPs.
• Group 3: RMGIC + 5 wt.% ZnO NPs.
• Group 4: RMGIC + 7 wt.% ZnO NPs.
• Group 5: RMGIC + 3 wt.% mesoporous ZnO NPs.
• Group 6: RMGIC + 5 wt.% mesoporous ZnO NPs.
• Group 7: RMGIC + 7 wt.% mesoporous ZnO NPs.

For the control group (Group 1), RMGIC powder (Fuji II LC Gold A2; GC Corp., Tokyo, Japan) was mixed with liquid at a ratio of 3.2:1 by weight (one scoop of powder to two drops of liquid), following the manufacturer's protocol.

For groups 2 through 7, the experimental powders were prepared by manually blending the appropriate weight percentages of either ZnO NPs or mesoporous ZnO NPs with the RMGIC powder. The mixed powders were placed into amalgam capsules and homogenized using a mechanical amalgamator (Ultramat 2; SDI Limited, Victoria, Australia) for 20 s. These modified powders were then mixed with Fuji II liquid to prepare each group's restorative material.

Micro shear bond strength (μSBS) and failure mode evaluation:An adhesive tape with a circular hole was applied to each specimen to define the bonding area. A polyvinyl chloride microtube (0.5 mm height, 0.7 mm inner diameter) was aligned with the hole [4, 19, 20]. The tube was filled with the RMGIC mixture, and a Mylar strip was placed on top [19]. Each specimen was cured for 40 s using an LED light-curing device (Blue Lex LD-105; Monitex, Taipei, Taiwan; 1500 mW/cm^2^, 440–480 nm), holding the curing tip 1 mm above the surface.

**Figure 2:**
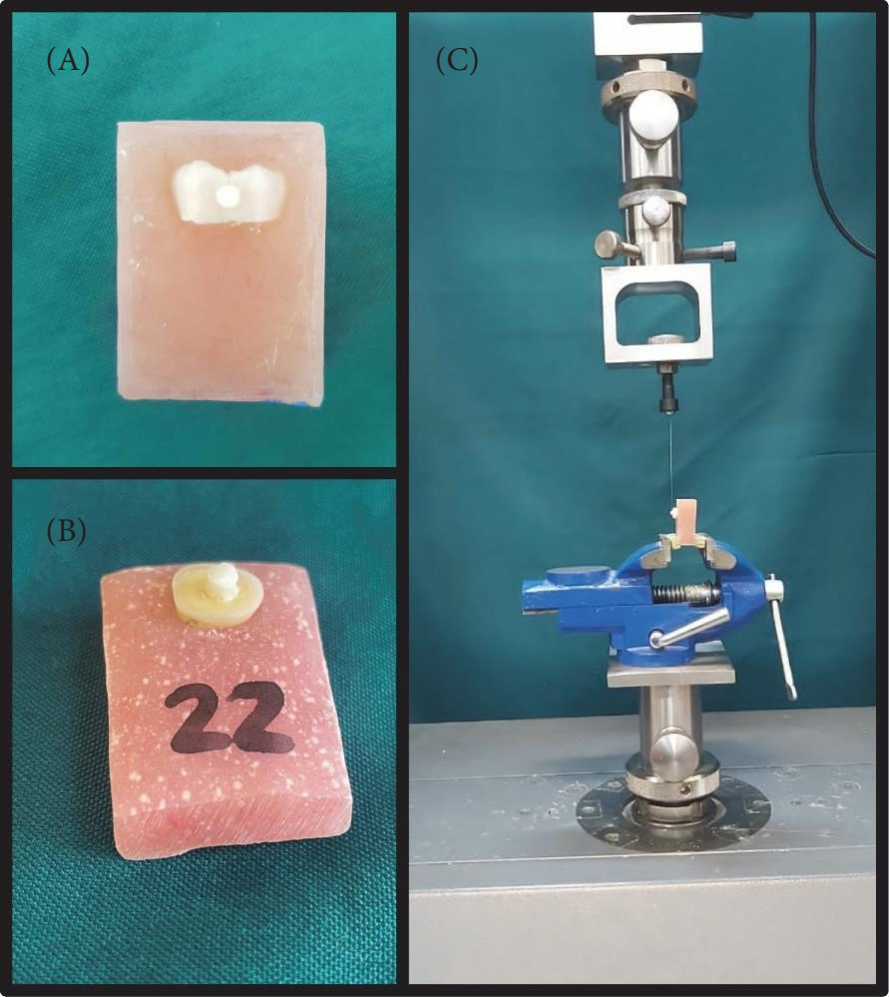
Setup for evaluating microshear bond strength. (A) Enamel sample after bonding resin-modified glass ionomer cement (RMGIC), (B) dentin sample following RMGIC bonding, and (C) specimen mounted in a universal testing machine, showing the RMGIC microcylinder secured with a stainless-steel ligature wire for load application.

The cured specimens were stored in distilled water at 37°C for 24 h before testing. Afterward, both the microtube and Mylar strip were carefully removed with a scalpel. Shear force was applied parallel to the bonding surface using a universal testing machine (Instron Z020; Zwick Roell, Ulm, Germany) at a crosshead speed of 1 mm/min. The force (*N*) at failure was recorded and divided by the bonding area to calculate bond strength in megapascals (MPa).  Figure 2 offers a stepwise visual depiction of the sample preparation and testing process. Part (A) shows an enamel specimen after bonding with RMGIC, while part (B) presents a similarly prepared dentin specimen, and part (C) illustrates the setup for the µSBS test, with the bonded specimen placed under a universal testing machine and a stainless-steel ligature wire looped around the RMGIC microcylinder for load application.

Each debonded surface was examined under a stereomicroscope (Carl Zeiss Inc., Oberkochen, Germany) at 40× magnification. Failure modes were categorized as:

• Adhesive: separation at the interface.
• Cohesive: fracture within the material or tooth substrate.
• Mixed: a combination of adhesive and cohesive failure [21].

### 2.4. Statistical Analysis

The dataset was first examined for normality using the Shapiro–Wilk test. Since, the distribution of values met the assumption of normality, the data were analyzed using a two-way analysis of variance (ANOVA) to determine the effects of two independent variables: the type of bonding surface (enamel or dentin) and the incorporation of NPs into the RMGIC.

To further investigate pairwise differences among the test groups, pair wised comparisons were performed using the Tukey's Honestly Significant Difference (HSD) test and independent *t*-test. All statistical evaluations were conducted with SPSS software (version 17.0; SPSS Inc., Chicago, IL, USA). The level of statistical significance was set at *p* < 0.05, with values below this threshold considered indicative of a significant difference.

## 3. Results

**Figure 3:**
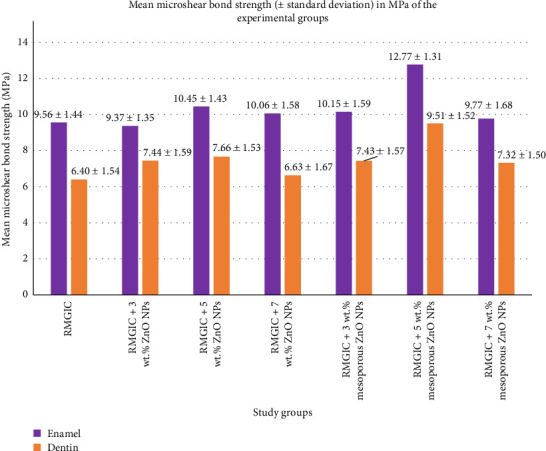
Bar chart displaying the microshear bond strength results, including standard deviations, for each experimental group. Abbreviations: RMGIC, resin-modified glass ionomer cement; ZnO NPs, zinc oxide nanoparticles.

A total of 140 specimens (10 samples per group) were evaluated for µSBS to enamel and dentin. The mean µSBS values (MPa) and their corresponding standard deviations (SD) for each subgroup are detailed in Table 1, with a graphical representation provided in Figure 3. The Shapiro–Wilk test confirmed the normal distribution of the data (*p* > 0.05), supporting the application of parametric statistical methods. Two-way ANOVA (Table 2) demonstrated significant main effects for both the type of bonding surface (enamel vs., dentin) and the incorporation of NPs into the RMGIC (both *p* < 0.001). Further, independent *t*-test analysis indicated that enamel exhibited significantly higher µSBS values than dentin (*p* < 0.001). However, no significant interaction effect was observed between the bonding substrate and NP incorporation (*p*=0.679).Tukey's post hoc test revealed that the subgroup containing RMGIC modified with 5% mesoporous ZnO NPs achieved the highest mean µSBS for both enamel and dentin, significantly outperforming all other groups (*p* < 0.001). No statistically significant differences were found among the remaining groups for either substrate (*p* > 0.05).

**Figure 4:**
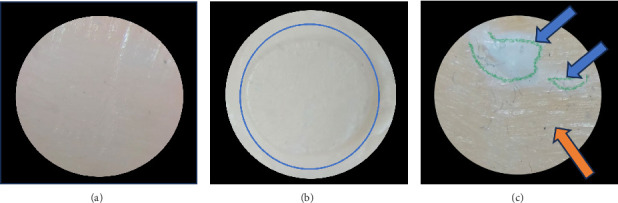
Representative fracture modes observed during the microshear bond strength test under 40× stereomicroscope magnification: (A) Adhesive failure, (B) cohesive failure within the resin-modified glass ionomer cement (RMGIC), with the fracture area circled in blue, and (C) mixed failure, showing cohesive failure in RMGIC (indicated by blue arrows and green outline) and adhesive failure at the interface (marked by orange arrows).

**Figure 5:**
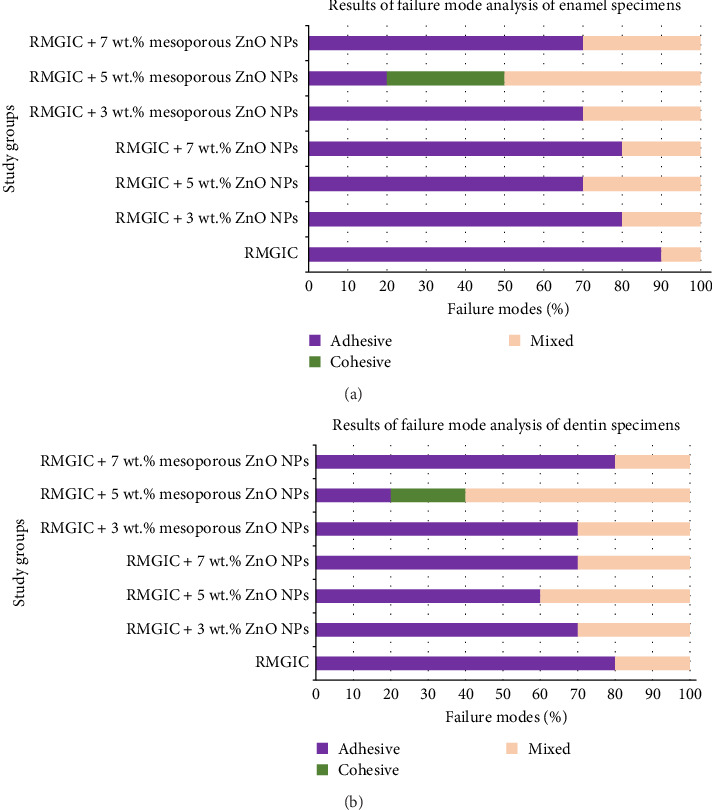
Bar chart illustrating the distribution of failure modes observed during the microshear bond strength test for all experimental groups. (A) Enamel specimens and (B) dentin specimens. Abbreviations: RMGIC, resin-modified glass ionomer cement; ZnO NPs, zinc oxide nanoparticles.

Representative images of the failure patterns observed during the μSBS testing are shown in Figure 4, while Figure 5 summarizes the distribution of failure modes across the experimental groups. Adhesive failure was the predominant mode in all groups, except for RMGIC containing 5 wt.% mesoporous ZnO NPs, where mixed failures were most common for both enamel and dentin. Cohesive failures within the RMGIC material were exclusively observed in this group, with three cases in enamel specimens and two cases in dentin specimens.

## 4. Discussion

This study evaluated the effect of incorporating ZnO and mesoporous ZnO NPs into RMGIC on μSBS to enamel and dentin. Both the type of substrate and NP modification significantly influenced bond strength, with enamel consistently showing higher values than dentin. Among the modified groups, RMGIC containing 5 wt.% mesoporous ZnO NPs exhibited the highest bond strength to both substrates, outperforming all other formulations.

The μSBS test is a reliable method for assessing adhesive bond strength, allowing multiple specimens from a single tooth and making it ideal for small bonding areas [22]. Unlike macroshear tests, which often suffer from uneven stress distribution and mixed loading, μSBS provides more accurate measurements by reducing these limitations. In macroshear tests, low applied loads can result in cohesive failures within the restorative material or tooth rather than at the adhesive interface [22]. Therefore, μSBS was used in this study to evaluate the effect of NPs incorporation on RMGIC bonding to enamel and dentin.

Secondary caries remains a common cause of GIC failure, highlighting the need for enhanced antimicrobial properties. To overcome this limitation, various antibacterial agents have been incorporated into GICs to improve their efficacy and longevity [9, 11].

Metal and metal oxide NPs have gained attention in dentistry for their antibacterial activity and ability to enhance restorative materials [9]. Their high bioactivity, biocompatibility, mechanical strength, and large surface-to-volume ratio make them ideal for improving both antimicrobial and physical properties of dental materials [10]. ZnO NPs are widely studied for their antibacterial efficacy, biocompatibility, stability, low toxicity, and affordability, with their antimicrobial activity largely attributed to their high surface area [10, 23].

Mesoporous materials have attracted interest in dentistry due to their antibacterial activity, drug-release potential, and biocompatibility [15, 24]. Mesoporous calcium-silicate NPs loaded with chlorhexidine (CHX) show strong antibacterial effects against *Enterococcus faecalis*, low cytotoxicity, controlled drug release, and remineralization properties, and are applied in bone repair and intracanal treatments [24]. Similarly, mesoporous silica NPs incorporated into resin composites effectively release CHX, inhibiting *S. mutans* and *Lactobacillus casei* without compromising material integrity [25].

Mesoporous ZnO NPs offer enhanced antibacterial activity, biodegradability, and drug-release capabilities, making them promising additives for RMGIC [15, 26]. ZnO also has a strong affinity for polyacrylic acid in RMGIC, which further supports its incorporation [27]. Additionally, adding 2 wt.% ZnO NPs to RMGIC offers antimicrobial benefits without affecting flexural strength or fluoride release [27]. Previous research has consistently emphasized that the effectiveness of restorative materials depends not only on their inherent strength but also on their ability to achieve durable adhesion to various tooth structures, with studies investigating strategies to enhance bond strength in different clinical scenarios [12, 28]. Building on this concept, the present study aimed to evaluate the effect of mesoporous ZnO NPs on the adhesive performance of RMGIC, ensuring that the antimicrobial benefits are achieved without compromising bond strength to enamel and dentin.

Mesoporous ZnO NPs exhibit stronger antibacterial activity than conventional ZnO NPs, largely due to their increased surface area and porous structure, which facilitate greater ion release and stronger interactions with bacterial cell membranes [15]. Their antimicrobial efficacy involves multiple synergistic mechanisms, including NP penetration that disrupts cell integrity, released Zn^2+^ ions that interfere with bacterial metabolism by inhibiting enzymes and nutrient uptake, and reactive oxygen species (ROS), such as hydroxyl radicals (•OH), superoxide anions (•O_2_^–^), and hydrogen peroxide (H_2_O_2_), which induce oxidative damage to bacterial membranes, proteins, and DNA [15, 29]. Together, these properties highlight the potential of mesoporous ZnO NPs to enhance both the antibacterial activity and clinical performance of RMGIC.

Enamel consistently showed higher bond strength than dentin across all groups, reflecting the influence of the bonding substrate on adhesive performance [20, 30]. This superior enamel bonding is attributed to its uniform, mineral-rich hydroxyapatite structure with high surface energy, which enhances wetting and micromechanical retention. In contrast, dentin's heterogeneous structure, with ~30% organic content and numerous dentinal tubules, reduces surface energy and hinders adhesive infiltration, explaining the lower bond strength values observed [30].

In this study, incorporating 5 wt.% mesoporous ZnO NPs into RMGIC significantly improved μSBS to enamel and dentin compared to the unmodified control, while other groups showed no significant differences. The enhanced performance likely stems from the nanoscale size of the NPs, which allows gap filling and mechanical reinforcement, and their large, well-organized mesoporous structure, which increases interaction with the resin matrix [31]. This mesoporous framework may also enable deeper resin penetration, strengthen the internal structure and improve stress distribution at the adhesive interface. Additionally, chemical affinity between ZnO and polyacrylic acid may enhance particle dispersion and material homogeneity, further supporting the observed bond strength improvements [27, 32].

The bond between RMGIC and tooth tissue involves both chemical adhesion and micromechanical interlocking [33]. Chemically, polyacrylic acid carboxyl groups form ionic bonds with calcium ions in enamel and dentin, while mechanically, the partially self-etching RMGIC allows resin monomers to infiltrate demineralized zones, creating a hybrid-like layer [34]. SEM and EDX analyses have shown intimate adaptation and ion exchange at the RMGIC–tooth interface, supporting long-term stability [34]. NPs can further enhance microstructure by filling voids and improving particle packing, strengthening micromechanical retention [35]. In particular, mesoporous ZnO NPs not only serve as reinforcing fillers within the RMGIC matrix but also release Zn^2+^ ions that may contribute to additional chemical interactions with enamel and dentin [36]. These ions can interact with phosphate groups in hydroxyapatite, potentially enhancing chemical adhesion to the mineral component of the tooth [36, 37]. In addition, Zn^2+^ ions can bind to type I collagen in the organic matrix, stabilizing it and reducing enzymatic degradation, thereby helping preserve the hybrid layer at the RMGIC-dentin interface [38]. Zinc ions promote collagen cross-linking, inhibit matrix metalloproteinases, and preserve the triple-helix structure, collectively maintaining dentin–resin integrity and hybrid layer stability [38–40]. Thus, mesoporous ZnO NPs may play a dual role, acting both as structural fillers and as active participants in the chemical bonding process, while their main effect remains reinforcing.

The study showed that increasing mesoporous zinc oxide nanoparticle (ZnO NP) concentration from 5 to 7 wt.% did not significantly improve bond strength. This may result from a saturation effect, where higher NP content leads to agglomeration and uneven dispersion, creating weak points that compromise the RMGIC structure. Excess NPs can hinder proper integration into the matrix, reduce the availability of Al^3+^ ions necessary for crosslinking with polyacrylic acid, and limit polymer network formation, all of which negatively affect bond strength [33, 41]. Similar findings have been reported in another study, where high NP concentrations caused agglomeration and weakened material properties [32]. These results highlight the need to maintain an optimal NP concentration to enhance antimicrobial activity, while preserving the structural integrity and adhesive performance of RMGIC.

The lack of improved bond strength with conventional ZnO NPs compared to 5 wt.% mesoporous ZnO NPs likely reflects their structural limitations. Conventional ZnO NPs have lower surface area and no organized pore network, reducing interaction with the RMGIC matrix and tooth surface, and limiting micromechanical interlocking and cohesive reinforcement. In contrast, mesoporous ZnO NPs offer greater surface area, better dispersion, and stronger integration with polyacrylic acid, enhancing internal structure and interfacial adhesion.

In this study, the conditioning step prior to RMGIC application was likely crucial in enhancing adhesion to enamel and dentin. The GC Cavity Conditioner (20% polyacrylic acid, 3% aluminum chloride hexahydrate, pH 1.8–2.0) efficiently removes the smear layer, increases surface wettability, and exposes tooth structure, promoting mechanical interlocking [42]. Conditioning with polyacrylic acid promotes the release and activation of calcium and phosphate ions in dentin, which are critical for chemical bonding with the polyalkenoic acid groups in RMGIC [42]. This process enhances surface contact and ion availability, strengthening the interaction between the restorative material and dental tissues. These effects likely contributed to the overall bond strength across all groups and may have further supported the superior performance of RMGIC containing 5 wt.% mesoporous ZnO NPs.

Failure mode analysis showed predominantly adhesive failures across most groups, consistent with the behavior of RMGICs. In contrast, the 5 wt.% mesoporous ZnO NP group displayed more mixed and cohesive failures, especially in enamel specimens. Such a shift indicates stronger interfacial adhesion, as these failures occur when interfacial strength exceeds the cohesive strength of the material, reflecting enhanced micromechanical and chemical interactions [43, 44]. The incorporation of mesoporous ZnO NPs likely produced a more homogeneous and reinforced matrix, supporting this effect. Cohesive and mixed failures are often correlated with higher bond strength values [43, 44]. Nevertheless, it must also be recognized that a predominance of cohesive failure could indicate internal weaknesses within the restorative material, with possible implications for long-term seal integrity and microleakage [45]. Thus, the increased incidence of cohesive and mixed failures in the 5 wt.% mesoporous ZnO NP group can be interpreted as evidence that interfacial adhesion was reinforced to the point of surpassing the material's own cohesive strength, rather than reflecting a weakening of the cement itself. Nevertheless, possible material-related limitations should still be considered in future studies.

This study demonstrates that incorporating 5 wt.% mesoporous ZnO NPs into RMGIC significantly enhances bond strength to both enamel and dentin. Incorporating mesoporous ZnO NPs not only improves RMGIC performance but also adds antimicrobial benefits, potentially reducing secondary caries. This study is among the first to systematically evaluate different NP concentrations on RMGIC bond strength, providing valuable insights for optimizing restorative materials for enhanced clinical outcomes.

This study presents some limitations. Only one commercially available RMGIC was tested. Moreover, the bond strength was evaluated after just 24 h of water storage, without assessing long-term durability, and thermocycling was not performed in this study; future research, including thermal cycling is recommended to assess the effect of thermal stress on the bond strength of RMGIC containing mesoporous ZnO NPs. The in vitro design cannot fully replicate the oral environment, including pH changes, masticatory forces, and abrasion. Additionally, despite careful weighing and blending of NPs, the in vitro study cannot fully confirm complete bio-dissolution or homogeneous integration of the added NPs within the RMGIC matrix. Future studies should test more materials and NP concentrations, include aging protocols, and evaluate additional mechanical and physical properties to better validate clinical performance.

## 5. Conclusion

Incorporation of 5 wt.% mesoporous ZnO NPs into RMGIC significantly enhanced the µSBS to both enamel and dentin compared with the unmodified control. Bonding to enamel consistently showed higher values than to dentin, underscoring enamel as a more favorable substrate for adhesion. Overall, the addition of mesoporous ZnO NPs improved bond strength and altered failure modes, suggesting their potential to reinforce the material and enhance interfacial performance. Nevertheless, these findings are limited to in vitro conditions. Future studies incorporating long-term aging, thermocycling, and in vivo assessments are needed to confirm the durability, biocompatibility, and clinical applicability of mesoporous ZnO NP–incorporated RMGICs.

## Figures and Tables

**Table 1:** The means (± standard deviations) of the microshear bond strength (μSBS) to enamel and dentin of the experimental groups.

Subgroup number	Experimental condition	Enamel	Dentin
1	RMGIC without any filler (control)	9.56 ± 1.44	6.40 ± 1.54
2	RMGIC + 3 wt.% ZnO NPs	9.37 ± 1.35	7.44 ± 1.59
3	RMGIC + 5 wt.% ZnO NPs	10.45 ± 1.43	7.66 ± 1.53
4	RMGIC + 7 wt.% ZnO NPs	10.06 ± 1.58	6.63 ± 1.67
5	RMGIC + 3 wt.% mesoporous ZnO NPs	10.15 ± 1.59	7.43 ± 1.57
6	RMGIC + 5 wt.% mesoporous ZnO NPs	12.77 ± 1.31	9.51 ± 1.52
7	RMGIC + 7 wt.% mesoporous ZnO NPs	9.77 ± 1.68	7.32 ± 1.50

Abbreviations: RMGIC, resin-modified glass ionomer cement; ZnO NPs, zinc oxide nanoparticles.

**Table 2:** Results of the two-way ANOVA test.

Source	Type III sum of squares	df	Mean square	*F*	*p*-Value
Bonding surface (enamel or dentin)	272.416	1	272.416	117.278	<0.001*⁣*^*∗*^
Incorporation of nanoparticles	130.134	6	21.689	9.337	<0.001*⁣*^*∗*^
Bonding surface × Incorporation of nanoparticles	9.246	6	1.541	0.663	0.679
Error	292.674	126	2.323	—	—
Total	11,757.765	140	—	—	—

Abbreviations: ANOVA, analysis of variance; df, degrees of freedom; F, f statistic.

*⁣* ^*∗*^Significant at *p* < 0.05.

## Data Availability

Data available on request from the author.
